# Exposure to household secondhand tobacco smoke and the odds of developing atopic dermatitis among adolescents: A causal mediation analysis

**DOI:** 10.18332/tid/176967

**Published:** 2024-02-01

**Authors:** Saeed Akhtar, Saad Al-Shanfari, Hussain Booalayan, Mosa Abdulrasool, Abdulwahab Boujarwa, Abdullah Al-Mukaimi, Omar Alkandery

**Affiliations:** 1 Department of Community Medicine and Behavioral Sciences, College of Medicine, Kuwait University, Safat, Kuwait

**Keywords:** asthma, tobacco smoking, atopic dermatitis, causal mediation analysis, household secondhand tobacco smoke exposure

## Abstract

**INTRODUCTION:**

Exposure to household secondhand tobacco smoke (SHS) among adolescents has been shown to be associated with atopic dermatitis, and affects disproportionality females. However, the mechanisms underlying this link are uncertain. This study sought to identify modifiable factors that mediate the relationship between household SHS exposure and atopic dermatitis among adolescents.

**METHODS:**

During October 2015, a cross-sectional study was conducted using the ISAAC questionnaire for data collection from adolescents enrolled in nine high schools of Hawalli – one of the six governorates of Kuwait. Data were collected on sociodemographic characteristics, self-reported tobacco smoking among adolescents, household SHS exposure (≥1 smokers at home vs none), self-reported asthma and atopic dermatitis. For causal mediation analysis an inverse odds-weighting approach was used.

**RESULTS:**

Of 746 participants, 74.8% were Kuwaiti, 50.1% were female, 12.4% were regular daily smokers and 54.1% had household SHS exposure, which was more common among Kuwaiti (79.6%) than non-Kuwaiti (20.4%) adolescents. The prevalences of self-reported asthma and atopic dermatitis were 20.6% and 14.9%, respectively. After adjusting for the pre-exposure covariates (i.e. sex and nativity), household SHS exposure had a significant (p=0.043) total effect, non-significant (p=0.133) natural direct effect, and marginally insignificant (p=0.058) natural indirect effect, which were jointly mediated by asthma status and adolescent’s self-reported smoking status, with a proportion of mediated risk to atopic dermatitis of 29.6%.

**CONCLUSIONS:**

Asthma and self-tobacco smoking among adolescents not only directly affected but also mediated household SHS exposure effect on atopic dermatitis risk. Voluntarily adopting a smoke-free home rule may minimize household SHS exposure, reduce the odds of developing asthma, and deter the initiation of tobacco smoking among adolescents. Such an effort will likely mitigate the atopic dermatitis risk among adolescents in this and other similar settings. If implemented, future studies may contemplate evaluating the impact of such intervention.

## INTRODUCTION

Tobacco smoking is a significant cause of global mortality and the single most prevalent risk factor worldwide. Furthermore, total tobacco-attributable deaths are projected to increase from 6.4 million in 2013 to 8.3 million by 2030^1^. There is substantial evidence that household secondhand tobacco smoke (SHS) exposure, among children and adolescents, has several ill-health consequences including an increased risk of sudden infant death syndrome, reduced lung growth, early development of cardiovascular diseases, increased propensity for respiratory infection, and the development of childhood asthma and atopic dermatitis^2^. The available epidemiological evidence on the relationship between household SHS exposure and allergic morbidity including asthma and atopic dermatitis among children and adolescents, is mainly based on the studies reported from Western countries^3^. Little published data on the link between household SHS exposure and allergic morbidity among adolescents are available from the Middle Eastern countries, including Kuwait. Previously, we have reported the prevalence of household SHS exposure in association with asthma and atopic dermatitis among adolescents in Kuwait^4,5^. However, the concurrent prevalence of asthma and atopic dermatitis, the nature of the interrelationship between these two allergic conditions, and demographics among the adolescents with household SHS exposure have not been explored. Here we aimed to examine the pathways from household SHS exposure to asthma, self-reported smoking, and atopic dermatitis after accounting for the effects of baseline covariates. Specifically, we sought to evaluate whether the association between household SHS exposure and atopic dermatitis was, to any extent, mediated through asthma, and adolescent’s cigarette smoking status, after accounting for the effects of pre-exposure covariates (i.e. sex and nativity) using a nonparametric inverse odds-weighting (IOW) approach for causal mediation analysis.

## METHODS

### Study design, setting and population

During October 2015, we conducted a cross-sectional study among high-school students in Hawalli – one of the six Governorates of Kuwait to: 1) assess the prevalence of household SHS exposure; 2) assess the prevalence of various allergic conditions including asthma, atopic dermatitis; and 3) evaluate the association between household SHS exposure and allergic morbidity including asthma and atopic dermatitis. The foremost contemplation in selecting this study population was their high expected frequency of household SHS exposure, its duration, and expected high prevalence of allergic morbidities. We obtained a list of schools located in Hawalli Governorate from the Ministry of Educations’ website, which included ten boys’ schools, nine girls’ schools in the public sector, and forty-five private schools with a co-education system. From this list, we selected nine schools as a sample of convenience, which included public-sector schools for boys (3), girls (3) and private-sector schools with co-education system (3). The students of either sex, of any nativity (Kuwaiti, non-Kuwaiti), currently enrolled in grade 11 or 12 at public or private-sector high schools, were eligible for inclusion in the study. The details of study design, setting, population and data collection have been previously described elsewhere^4,5^.

### Questionnaire

A structured and self-administered questionnaire was developed in English for the collection of data on sociodemographic characteristics, self-reported smoking status, and household SHS exposure, as described elsewhere^4,5^. The standardized international study of asthma and allergies in childhood (ISAAC) core questionnaire was used for diagnosis of asthma and atopic dermatitis^6^. In this study, a respondent was regarded as an asthmatic, if during the past 12 months, he or she reported to have had four or more episodes of wheezing, or one or more episodes of wheezing with the use of an inhaler, or ever diagnosed as asthmatic by a physician^6^. Moreover, a respondent was considered to have had atopic dermatitis, if he or she had recurrent itchy rash for at least 6 months, and localization at the folds of the elbows, behind the knees, in front of the ankles, or around the neck, or diagnosed to have had atopic dermatitis by a physician^6^. For the diagnosis of asthma and atopic dermatitis among children and adolescents, the estimates of diagnostic parameters of the ISAAC questionnaire including sensitivity (≥87.5%), specificity (≥80.4%), internal consistency (Cronbach’s alpha=0.81)^7^, and reliability (Kappa statistic range=0.81–0.85) have been reported earlier^8,9^. The questionnaire was initially developed in English and the final version was also translated into Arabic for actual use. The questionnaire was pre-tested on 20 students similar to our potential study participants and modifications were made as needed. The final questionnaire comprised 21 questions, and on average took five minutes for its completion^4,5^.

### Data collection

We intended to enroll about 100 senior students from grades 11 and 12 of the selected schools. We approached and sought the help of the teacher in-charge of the class for completion of the questionnaire by the students at the end of the class. The study aims were shared with the students by the respective class teachers, and were informed that their study participation was voluntary. Moreover, the students were assured of the confidentiality of the collected information. Afterwards, consenting students were invited to complete the written consent form attached to the study questionnaire. We adopted a similar procedure for data collection, both in public-sector and private schools. For this cross-sectional study, we estimated a sample size of 827 students to assess the prevalence of self-reported allergic morbidity, including asthma and atopic dermatitis, at a 95% confidence level (CI), with 2.5% bound on the error of estimation assuming a prevalence of allergic morbidity of 16% in our target population^10,11^. The computed sample size was increased to 850 students to account for any potential refusals.

### Data analysis

**Figure 1:**
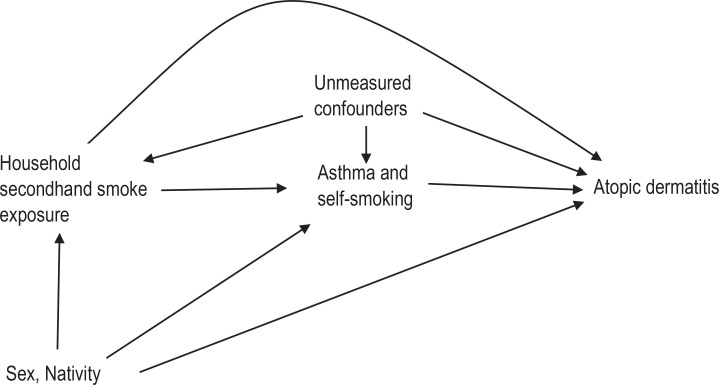
Directed acyclic graph for hypothesized causal mediation analysis of household secondhand tobacco smoke exposure effect (E) on the atopic dermatitis (Y) risk mediated through asthma, and self-reported smoking status (mediators) conditional on sex, and nationality (pre-exposure covariates) among adolescents in Kuwait, October 2015 (N=747)

To characterize the study sample, descriptive statistics including mean with standard deviation (SD) for quantitative variables, and frequencies and proportions (%) for categorical variables, were computed. The prevalence (%) of household SHS exposure, allergic morbidities, including asthma and atopic dermatitis, were computed. Previously, we have presented elsewhere the description of statistical methods used for univariable and multivariable analyses of associations between sociodemographics, risk factors and atopic dermatitis, as well as for asthma^5^. All the statistical tests were two-tailed at a prespecified level of significance (α=0.05). For causal mediation analysis, the directed acyclic graph (Figure 1) depicts the assumed causal relationships between household SHS exposure (exposure), self-reported smoking status, asthma status (mediators), sex, nativity (pre-exposure confounders) and the atopic dermatitis status (outcome). The total effect (TE) of household SHS exposure among adolescents was decomposed to natural direct effect (NDE) and natural indirect effect (NIE), with two mediators (asthma status, self-reported smoking status) conditioned on two pre-exposure covariates (sex, nativity). In this study, the NDE contrasts the odds of atopic dermatitis among adolescents with household SHS exposure and adolescents without household SHS exposure; if for all adolescents, the level of asthma status and self-reported smoking status were set to levels that would have been observed had the adolescent been without household SHS exposure. The NIE contrasts the odds of atopic dermatitis among adolescents with household SHS exposure with their observed level of asthma status and self-reported smoking status to the counterfactual odds, where levels of asthma status and self-reported smoking status were set to levels that would have been observed had the adolescents with household SHS exposure been without household SHS exposure. NDE and NIE were averaged over all adolescents, including asthmatic and non-asthmatic, smokers and non-smokers) and were also evaluated at mean population levels of the covariates, including sex and nativity.

To estimate the NDE and NIE, we conducted a nonparametric causal mediation analysis using the IOW method within a counterfactual framework, which is more robust against model misspecification such as interaction and nonlinearity^12^. Furthermore, when multiple mediators are considered concurrently, this is an appropriate approach, which takes advantage of the invariance property of the odds ratio. IOW synthesized information on the odds ratio (OR) between household SHS exposure, self-reported smoking status and asthma into a weight, which was then used to estimate NDE via a weighted regression analysis of atopic dermatitis. Subsequently, NIE is estimated by subtracting NDE from the TE obtained from the atopic dermatitis model without weights. The 95% CI for the parameters’ estimates were obtained by nonparametric bootstrapping with 1000 replications. The proportion of the TE jointly mediated by two mediators was computed for the model employed. We did not explicitly carry out power calculation for the mediation analysis, mainly because current literature on this topic is fairly limited, and, so far, no specific method is available^13^. However, it has been argued that for a single-level mediation analysis using the percentile bootstrap method, a sample size of 558 gives 80% power to estimate efficient NIE and NDE^14^. In this current evaluation with a given sample size, we hope to have a somewhat larger power than 80% for the estimation of NIE and NDE.

The validity of the nonparametrically computed NDE and NIE from the observational data depends on the identification assumptions, i.e. there are no unobserved confounding effects for the association of: 1) exposure-mediators, 2) mediators-outcome, or 3) exposure-outcome, conditional on the pre-exposure confounders. An additional requisite assumption was that there were no confounding variables of the mediator-outcome relationship affected by the exposure. To evaluate the robustness of the results to the presence of unobserved confounding, sensitivity analysis was carried out^15^. In the sensitivity analysis, estimates of NDE and NIE under the given level of unobserved confounding were obtained. Sensitivity parameters’ estimates were used to quantify the influence of potential unobserved confounders. The purpose was to estimate the effects that would have been obtained had the unobserved confounders’ effects been accounted for. Missing values were handled by list-wise deletion, and p<0.05 was considered statistically significant for all analyses. The study protocol was approved by the Kuwait University Health Sciences Center Ethical Committee(# 1958/10/11/2015). Formal permission was sought from the Ministry of Education to conduct this study at public-sector schools and from the principals of selected private-sector schools. The study adheres to the Strengthening the Reporting of Observational Studies in Epidemiology (STROBE) guidelines^16^.

## RESULTS

**Figure 2:**
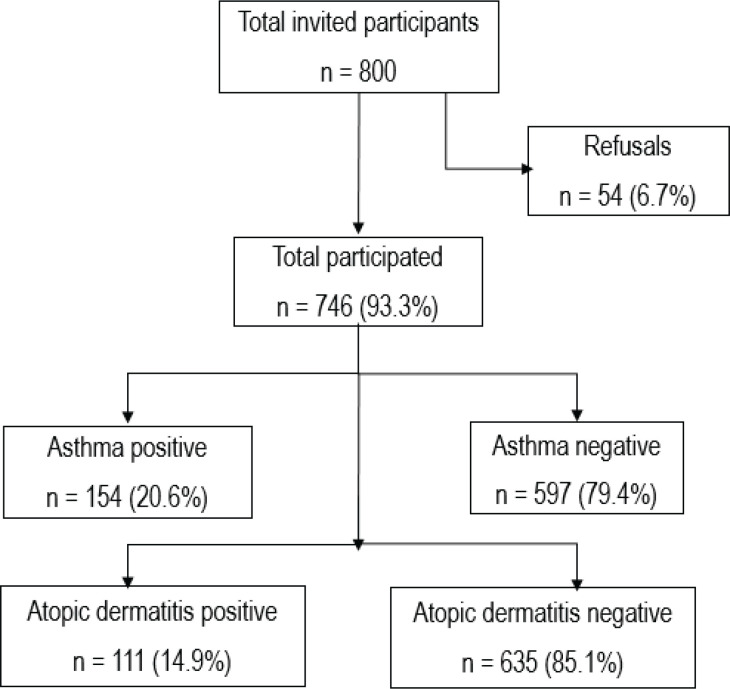
Flow-chart for participants enrollment for the study to conduct the hypothesized causal mediation analysis of household secondhand tobacco smoke exposure effect (exposure) on the atopic dermatitis (outcome) risk mediated through asthma and self-reported smoking status (mediators) conditional on sex, and nationality (pre-exposure covariates) among adolescents in Kuwait, October 2015 (N=747)

Of the invited 800 students, 746 (93.3%) participated and completed the study questionnaire. The distributions of age and sex of the non-respondents were similar to those of the respondents. The main reasons for non-participation cited by the non-respondents were ‘no interest’ (n=30; 0.04%) or ‘do not have time’ (n=24; 0.03%). A flow chart shows the participant's enrollment and retention in the study (Figure 2). Of 746 participants, 74.8% were Kuwaiti, 50.1% were female, 12.4% were regular daily smokers, and 54.1% had household SHS exposure. The household SHS exposure was more common among Kuwaiti (79.6%) than non-Kuwaiti (20.4%) adolescents. The prevalence of self-reported asthma and atopic dermatitis in the study sample of adolescents was 20.6% and 14.9%, respectively. The distributions of other characteristics are given in Table 1.

**Table 1:** Sociodemographics, prevalences of cigarette smoking, household secondhand tobacco smoke (SHS) exposure, asthma and atopic dermatitis among school-going adolescents, October 2015, Kuwait (N=746)

*Characteristics*	n*	*%*
**Sociodemographic**		
**Type of school**		
Government	589	79.0
Private	157	21.0
**Age** (years), mean (SD)	16.78 (0.68)	
**Gender**		
Male	372	49.9
Female	374	50.1
**Nationality**		
Kuwaiti	552	74.8
Non-Kuwaiti **	186	25.2
**Monthly income** (KWD)		
<500	32	4.7
500–1000	126	18.3
1001–1500	144	20.9
1501–2000	105	15.3
>2000	281	40.8
**Smoking exposure and disease**		
**Current smoking status**		
Yes	91	12.4
No	643	87.6
**Smoking duration** (years)		
<1	13	14.6
1–2	21	23.6
>2	55	61.8
**Number of smokers at home**		
None	338	45.9
One or more	398	54.1
**ETS exposure at public places** (hours/week)		
<3	336	47.7
3–6	205	29.1
6–9	75	10.6
>9	89	12.6
**Wheezing ever**		
Yes	197	26.2
No	554	73.8
**Wheezing in the last 12 months**		
Yes	151	20.1
No	600	79.9
**Physician-diagnosed asthma**		
Yes	123	16.4
No	628	83.6
**Self-reported asthma status**		
Yes	154	20.6
No	597	79.4
**Atopic dermatitis status**		
Yes	111	14.9
No	635	85.1

* Total of categories may not add up to the total sample size (N=746) due to missing values.

** Non-Kuwaiti adolescents were of Egyptian, Syrian, Indian, Pakistani, etc. nationalities.

KWD: 1 Kuwaiti Dinar about US$3.26.

Household SHS exposure had a significant TE on the likelihood of atopic dermatitis (OR_TE_=1.57; 95% CI: 1.01–2.42, p=0.043) among adolescents. After accounting for the influences of the pre-exposure covariates, causal mediation analysis using the IOW approach showed that SHS exposure at home had a non-significant NDE (OR_NDE_=1.40; 95% CI: 0.90–2.17, p=0.133), marginally significant NIE (OR_NIE_=1.12; 95% CI: 1.00–1.26, p=0.058) jointly mediated through asthma and self-reported smoking status with a proportion of 29.6% mediated effect on the likelihood of atopic dermatitis (Table 2).

**Table 2:** Causal mediation analysis* to estimate the total, direct and indirect effects of household secondhand tobacco smoke (SHS) exposure on atopic dermatitis risk among school-going adolescents, October 2015, Kuwait

*Household SHS exposure*	*Atopic dermatitis risk*		
	*OR*	*95% CI*	*p*
Natural indirect effect	1.12	1.00–1.26	0.058
Natural direct effect	1.40	0.90–2.17	0.133
Total effect	1.57	1.01–2.42	0.043

* Household secondhand tobacco smoke (SHS) exposure (Exposure). Adolescent’s self-reported cigarette smoking status (Mediator). Adolescent’s asthma status (Mediator). Adolescent’s atopic dermatitis status (Outcome). Sex, nationality (Pre-exposure covariates).

Sensitivity analyses showed that for any unobserved confounder to explain away the observed parameter estimates of TE, NDE, and NIE of household SHS exposure on the likelihood of atopic dermatitis, it should amplify the household SHS exposure effect on atopic dermatitis by relatively much larger TE (OR_TE_=2.52; 95% CI: lower limit 1.11), NDE (OR_NDE_=2.15; 95% CI: lower limit 1.80), and NIE (OR_NIE_=1.49; 95% CI: lower limit 1.00) (Table 3).

**Table 3:** Sensitivity analysis for unobserved confounding parameters’ estimates and 95% lower limit obtained in causal mediation analysis to estimate the total, direct and indirect effects of household secondhand tobacco smoke on atopic dermatitis risk among adolescents using inverse odds ratio-weighting method

*Atopic dermatitis*	*Odds ratio*	*95% confidence interval lower limit*
Indirect effect	1.49	1.00
Direct effect	2.15	1.80
Total effect	2.52	1.11

Household secondhand tobacco smoke (SHS) exposure (Exposure); Adolescent’s self-cigarette smoking status (Mediator). Adolescent’s asthma status (Mediator); Adolescent’s atopic dermatitis status (Outcome); Sex, nativity (Pre-exposure covariates).

## DISCUSSION

Household SHS is one of the most common air pollutants. Several morbidities, including those of respiratory and cardiovascular systems are attributed to household SHS exposure. Children and adolescents are more likely to suffer from household SHS exposure-related adverse health effects^4,17^. In Kuwait, relatively recently, household SHS exposure has been reported to be as high as 39%^18^ and 45%^19^. We have previously reported a high prevalence of self-reported asthma (20.5%), physician-diagnosed asthma (16.4%), and atopic dermatitis (14.9%) among adolescents in relation to household SHS exposure^4,5^. However, there is a lack of understanding of different mechanisms that explain the observed impact of household SHS exposure on the atopic dermatitis risk, which has a relevance to evidence-based design and implementation of voluntary adoption of smoke-free home rule.

This study aimed to identify the modifiable factors that mediate the effect of household SHS exposure on atopic dermatitis risk among adolescents. The results showed that though NIE of household SHS exposure on atopic dermatitis was marginally significant, yet about 30% of the TE was jointly mediated by asthma and adolescents’ self-reported smoking status. To the best of our knowledge, this is the first study which has undertaken a causal mediation analysis to quantify the NIE of household SHS exposure on the risk of atopic dermatitis among adolescents. Previously, however, a local and a regional study reported an independent relationship of sex, passive smoking at home and asthma, with atopic dermatitis status among adolescents^10,20^. In the later study, it was demonstrated that, after adjusting for the effects of other lifestyle factors, adolescents were significantly more likely to have suffered from atopic dermatitis if they were female or asthmatic, indicating a direct effect of nearly the same magnitude as observed in the present study^20^. Another study from the region found that adolescents with atopic dermatitis were significantly more likely to be asthmatic^21^. A study from Lebanon showed that a mother’s waterpipe smoking was significantly associated with atopic dermatitis with a TE, which was somewhat higher than the TE reported in the present study^3^.

In observational studies, there is a risk of unmeasured confounding. We conducted a sensitivity analysis to assess the effect of a potential violation of this assumption of no unmeasured confounding of the relationship between mediators (asthma status, self-reported smoking status) and outcome (atopic dermatitis status) but also the associations involving exposure (household SHS exposure)^15^. The results of sensitivity analysis showed what would have been the magnitudes of various effects had the unobserved confounding effect adjusted for modeling TE, NDE and NIE. Therefore, to explain away the observed TE, NDE, and NIE of household SHS exposure on atopic dermatitis risk by any unobserved confounder, it should magnify the household SHS exposure effect on atopic dermatitis risk by relatively much larger magnitudes of these observed effects, which appeared to be unlikely.

### Strengths and limitations

This is a novel study reporting causal mediation analysis of NIE of household SHS exposure through two modifiable mediators, i.e. asthma and self-reported smoking status, along with the NDE and TE on atopic dermatitis risk among adolescents. However, the following limitations of this study should be considered in the interpretation of the results. First, the study participants were enrolled as a sample of convenience; therefore, generalizability of the results needs to be exercised with care. Second, we could not account for the exact number of smokers at home, including the relationship of smokers with the adolescents in the study. These variables could have differential effects on the household SHS exposure dose and the two mediators, i.e. asthma status, and self-reported smoking status. Future studies may evaluate the dose-response assessment of this exposure. Third, the data collection was undertaken using a cross-sectional study design; therefore, temporal ordering of exposure, mediators and outcome was difficult to determine. Therefore, causal inference needs to be considered with care. Fourth, the data for this study were collected back in October 2015. Therefore, the magnitudes of the disease conditions (i.e. asthma, atopic dermatitis) and/ or risk factors (i.e. active tobacco smoking or household SHS exposure) might have undergone temporal variation. However, a recent study among university students reported the prevalence of active smoking as 22.4%, which is substantially and understandably more than the 12.4% recorded in our study. The age difference between the participants in the two studies seems to have contributed to the variation in the prevalence of active smoking recorded in the two studies. Nevertheless, the prevalences of household SHS exposure (54.1% vs 48.9%), self-reported asthma (20.6% vs 20.0%) and atopic dermatitis (14.9% in both studies) were about the same as reported in our study and the recent study^19^. Finally, most of the measured variables were based on recall; however, since participants were adolescents, it is expected that they have good recall ability, with minimal recall bias in the measurement of study variables.

## CONCLUSIONS

This study showed that asthma status and self-reported smoking not only directly affect the atopic dermatitis risk, but they also mediate the effect of household SHS exposure on the likelihood of atopic dermatitis among adolescents. Hence, voluntarily adopting a smoke-free home rule may not only minimize the household SHS exposure and reduce the odds of developing asthma, but also discourage the initiation of smoking among adolescents in this and other similar settings. Future studies may contemplate evaluating such an intervention.

## Data Availability

The data supporting this research are available from the authors on reasonable request.
